# EDITORIAL: Advances in Dystonia

**DOI:** 10.2174/157015913804999496

**Published:** 2013-01

**Authors:** Cristopher Bragg, Nutan Sharma

Last year an unprecedented number of dystonia researchers, clinicians, and patient advocates from around the world came together in Barcelona for the 5^th^ International Dystonia Symposium. It was arguably the largest event of its kind, dedicated solely to presenting the latest research on the dystonias, a diverse group of movement disorders that all involve a debilitating loss of motor control. The conference was the culmination of a tremendous effort put forth by many individuals, including members of the European Dystonia Federation, the Dystonia Medical Research Foundation, the Dystonia Coalition, as well as dedicated international and local organizing committees. For three days, the record number of attendees heard and discussed the latest advances in the field, encompassing a wide range of basic science and clinical topics. The event was widely heralded as a resounding success, demonstrating to all in attendance the accomplishments that have been made in understanding and treating dystonia.

In assembling this special issue of *Current Neuropharmacology*, our goal was to highlight some of the same topics considered to be of particular interest at last year’s symposium. Because multiple, excellent reviews have recently summarized progress relating to primary early onset generalized dystonia, such as DYT1, we have largely emphasized discussions of nonprimary and focal dystonias here.

Casper and colleagues provide a broad overview of disorders in which dystonia appears as a prominent feature alongside other deficits. Although these “primary-plus” syndromes and secondary dystonias reflect seemingly disparate underlying defects, the authors note recent evidence implicating common disturbances in dopaminergic neurotransmission, cellular stress responses, and signaling within the cerebellum and basal ganglia. Schneider and colleagues in turn focus on a specific type of secondary dystonia: the syndromes classified as Neurodegeneration with Brain Iron Accumulation (NBIA). They present a growing list of NBIA-related gene mutations and describe the heterogeneous clinical phenotypes which may result. Together these two reviews cover considerable ground, demonstrating how diverse molecular defects within the central nervous system can lead to different clinical syndromes that share dystonia as a common feature.

While the most prevalent forms of dystonia may well be the adult-onset focal disorders, the underlying mechanisms remain poorly understood. Three of the included reviews consider different aspects of these syndromes. Chang and Frucht provide a comprehensive survey of focal dystonias that develop in musicians, describing the various clinical manifestations and current treatment options. The report by Evinger focuses specifically on benign essential blepharospasm and, in particular, the animal models currently available for probing the neural circuitry associated with eyelid dystonia. Finally, the review by Blood takes a systems-level approach, integrating findings from brain imaging studies of focal dystonias that may point towards a common pathophysiology.

The report by Elbe deals with a different yet highly significant clinical matter: the relationship between dystonia and tremor. The author summarizes the published clinical criteria commonly used to classify tremor, while outlining numerous issues that often complicate its distinction from dystonia. The review further posits that the current classification scheme for essential tremor may place too much emphasis on the often difficult task of excluding other clinical signs. A more useful approach may be an alternative, broader classification for “primary” (rather than essential) tremor, one that focuses more on the identification of underlying causes rather than narrowly defined exclusion criteria.

The remaining contribution speaks to the critical issue of drug discovery in dystonia. Caldwell and colleagues describe recent advances in the development of invertebrate models of dystonia that can serve as powerful tools for small molecule screening and target validation. Given that current pharmacologic therapies for dystonia remain limited, the need for such tools is extremely high.

While it was clear from the work presented in Barcelona last year that significant progress has been made in the general field of dystonia, it is still equally apparent that much remains to be done. For many patients, current treatment options are insufficient and provide only limited, symptomatic relief. For most forms of dystonia, the affected cellular pathways and disease mechanisms are still not known; and while many dystonia subtypes show evidence of heritability, in most cases the causative gene mutations have yet to be elucidated. Without a better understanding of the underlying pathobiology, developing assays to screen for candidate therapeutics has been difficult. Yet despite these caveats, there is great cause for optimism. The record attendance at last year’s symposium demonstrated how the dystonia field continues to expand, with new investigators bringing fresh perspectives to these issues. Our hope is that the articles presented here will catalyze further investigations into the pathophysiology of dystonia and that such efforts may one day offer the insight needed to design new treatment strategies.

